# Structural and Functional Characterization of Porcine Adeno-Associated Viruses

**DOI:** 10.3390/v17091260

**Published:** 2025-09-18

**Authors:** Austin Nelson, Mario Mietzsch, Jane Hsi, Julia Eby, Paul Chipman, Robert McKenna

**Affiliations:** Department of Biochemistry & Molecular Biology, University of Florida, Gainesville, FL 32610, USA

**Keywords:** adeno-associated virus, porcine AAV capsids, cryo-electron microscopy, gene therapy, neutralizing antibodies

## Abstract

Current gene therapy treatments utilizing adeno-associated virus (AAV) vectors are based on capsids of primate origin. However, pre-existing neutralizing anti-AAV antibodies, that are present in a significant portion of the population, can lead to vector inactivation and reduced therapeutic efficacy. Advances in DNA sequencing have facilitated the discovery of many AAVs from non-primate species, including isolates from pigs, which exhibit up to 50% capsid protein sequence divergence, compared to primate AAV serotypes. In this study, AAVs isolated from porcine tissues (AAVpo.1 and AAVpo.6) were selected for structural characterization due to their low capsid protein VP1 sequence identity compared to each other and to AAV9. The AAV vectors were produced via the standard triple transfection system in HEK293 cells using AAV2 *rep* to package AAV2-ITR vector genomes and were purified by iodixanol density gradient ultracentrifugation. The capsid structures of AAVpo.1 and AAVpo.6 were determined using cryo-electron microscopy and then compared to each other in addition to the AAV5 and AAV9 structures. Given that porcine AAVpo.6 has been reported to infect human cells and the ability to cross the blood–brain barrier, the functional characterization was focused on the identification of a potential glycan receptor utilized by the porcine capsids. Additionally, the porcine AAV capsid reactivity to human derived anti-AAV antibodies was assessed to evaluate the potential for these capsids to be used as alternative vectors for gene therapy, particularly for patients with pre-existing immunity to primate-derived AAV serotypes.

## 1. Introduction

The adeno-associated viruses (AAVs) are members of the *Parvoviridae*, a family of non-enveloped, linear single-stranded DNA viruses. They are assigned to the subfamily *Parvovirinae* and genus *Dependoparvovirus* and are found in a wide range of vertebrates, including humans, pigs, cattle, birds, bats, and reptiles [1]. The ~4.7 kb ssDNA genomes of the AAVs contain inverted terminal repeats (ITRs) flanking two main open reading frames, the *rep* and *cap* genes [2]. The ITRs and proteins generated from the *rep* gene are involved in the processes of genome replication and packaging [3]. The *cap* open reading frame encodes three viral proteins (VP), VP1, VP2, and VP3, that form the capsid shell. They have overlapping sequences at their C-termini so that VP3 (~60 kDa) is completely contained in VP2 (~67 kDa), and VP2 is entirely contained in VP1 (~82 kDa) [4].

The AAV capsid is comprised of a total of 60 VP monomers. On average five copies of VP1 and VP2 are incorporated into the capsid, with the remaining 50 monomers VP3, resulting in the typical ratio of 1:1:10 ratio for VP1, VP2, and VP3 [5,6]. However, the incorporation of these proteins is stochastic, creating significant heterogeneity among individual capsids [7]. The structures of the several AAV capsids have been determined by X-ray crystallography and/or cryo-electron microscopy (cryo-EM) [8,9]. All AAV capsids exhibit T = 1 icosahedral symmetry, but only the shared C-terminal VP3 region has been resolved, while the extended N-termini of VP1 and VP2 are intrinsically disordered [10]. Within VP3, the central core motif is an eight-stranded β-barrel (jellyroll motif) formed by two sets of four antiparallel β-sheets (βBIDG and βCHEF), with an additional βA-strand and one α-helix [5]. Loops connecting these β-strands form the capsid surface and vary among AAVs, which have been designated as variable regions (VRs): VR I–IX [11,12]. These variations are the result of the evolution of the capsid surface to promote host entry and cell trafficking to the nucleus where it delivers its genome [13]. Thus, the capsid surface determines the cell and tropism as well as antigenicity [14,15]. Despite these differences, the overall structural architecture of the capsid is conserved across the AAVs, including the 5-fold pore, 3-fold protrusions, 2/5-fold wall, and a 2-fold depression [16].

The AAVs are being utilized to treat monogenic disorders through targeted gene delivery [17]. Examples of successful AAV-based therapies include Luxturna for Leber’s Congenital Amaurosis and Zolgensma for spinal muscular atrophy [18,19]. Although much progress has been made in recent years for AAV-based gene therapies, the neutralization of the capsid, by pre-existing antibodies, remains a major hurdle [20]. These antibodies are the result of naturally circulating AAVs. The exposure to these viruses activated the host’s adaptive immune system to generate neutralizing antibodies (NAbs) to prevent future infections [21]. The resulting seropositivity rates for various AAV serotypes in the human population range from 40 to 70% [22]. Hence, when the same or similar AAV vector is used in a treatment, it can be neutralized by these Nabs, and seropositive individuals are typically excluded from AAV clinical trials and receiving these therapeutics [23,24].

One possible solution to evade this pre-existing immunity is to utilize AAV capsids for transgene delivery that do not disseminate in the human population. Such capsids range from primate AAVs that are closely related to human variants to more phylogenetically distant AAVs, such as quail adeno-associated virus [25,26]. AAVs from bats and bovines have also been characterized for their potential as AAV vectors [27,28,29]. Porcine AAVs, in particular, have shown promise, demonstrating the ability to cross the blood–brain barrier (BBB) in mice, transduce human cell lines like HEK293, and evade neutralization by human sera [30]. Pigs are considered strong candidates for immunology research and organ transplantation due to their anatomical and physiological similarities to humans [31]. In 2009, several porcine AAVs were isolated from various pig tissues, with AAVpo.1 detected in the widest range of tissues [32]. A recombinant AAV2/AAVpo.1 vector has been tested in mice, showing greater responsiveness in skeletal muscle compared to lung and liver. It also demonstrated transduction in murine and porcine retinal cell lines [32]. When tested against pooled human antibodies, AAV2/AAVpo.1 showed no neutralization, unlike AAV2 and AAV5. In 2014, additional porcine AAVs, including AAVpo.4 and AAVpo.6, were isolated and found to cross the BBB in mice [30]. AAVpo.6 was able to transduce mouse lung tissues but was partially neutralized by human antibodies.

As AAV vectors capable of evading human neutralizing antibodies emerge, non-human based AAVs present a promising alternative. AAVpo.1 and AAVpo.6 efficiently transduce HEK293 cells, package AAV2-based transgenes, and exhibit unique VR region structures. AAVpo.6 crosses the BBB in pigs, a trait of significant interest for gene therapy. Their potential as therapeutic vectors depend on assessing whether human NAbs cross-react with porcine AAVs. Identifying viral surface variants that enable antibody evasion or alter human cell transduction is critical for future capsid engineering.

Presented here are the cryo-EM structures of AAVpo.1 and AAVpo.6, their glycan binding analysis, and interactions with human NAbs derived from patients post-AAV9 exposure. Insights from these studies may help to guide the development of AAV vectors, improving existing therapies and expanding patient accessibility.

## 2. Materials and Methods

### 2.1. Cloning of the Recombinant Porcine AAVs

The entire AAVpo.1 and AAVpo.6 *cap* genes were synthesized (GeneWiz, South Plainfield, NJ, USA) using the GenBank accession numbers FJ688147 and JX896664, respectively. Subsequently, both capsid genes were amplified by standard PCR with the following primers: Fwd: 5′-AAATGATTTAAAGTAGGCATGTC-3′ and Rev: 5′-CGGTTTATTGATTAACTCTAGATTACAG-3′. Similarly, a plasmid containing the AAV2 *rep* and AAV5 *cap* gene [33] was amplified using the primers: Fwd: 5′-TAATCTAGAGTTAATCAATAAACCGGTTGATTCGTTTCAGTTG-3′ and Rev: 5′-GACATGACTACTTTAAATCATTTATT-GTTCAAAGATGCAG-3′. The DNA fragments were reassembled utilizing a Gibson Assembly Kit (New England Biolabs, Ipswich, MA, USA) according to the manufacturer’s standard protocol which replaced the AAV5 *cap* gene of the original plasmid with the cap gene of the porcine AAVs. The correct assembly of the recombinant AAVpo.1 and AAVpo.6 plasmids were confirmed by Sanger sequencing (GeneWiz, South Plainfield, NJ, USA).

### 2.2. Production and Purification of the Porcine AAVs

Recombinant AAVs packaging a luciferase gene were produced using the standard triple transfection system in HEK293 cells utilizing pTR-UF3-Luciferase [33], pHelper (Stratagene, La Jolla, CA, USA), and the AAV2-*rep*-AAVpo.1-*cap* or AAV2-*rep*-AAVpo.6-*cap* plasmid. HEK293 cells were maintained in DMEM with 10% FBS and 1% antibiotic antimycotic solution (Thermo Fisher, Waltham, MA, USA) at 37 °C with 5% CO_2_. 72 h post-transfection, cells were harvested via centrifugation at 2000 RPM for 10 min at 4 °C, and cell pellets were resuspended in 1X PBS buffer (supplemented with 2.5 mM KCl and 1 mM MgCl_2_). The cell pellets were lysed via three freeze–thaw-vortex cycles, treated with benzonase (0.1 µL/mL) for 1 h at 37 °C, and clarified using NaCl (80 µL/mL) and centrifugation at 7000 RPM for 10 min at 4 °C. The final supernatant was collected and stored at −80 °C prior to purification. Genome-containing particles were purified via iodixanol density gradient centrifugation as described previously [34]. Western blots were run to identify all AAV-containing fractions, and quantitative PCRs for genome-containing particles. Selected fractions were pooled, concentrated, and buffer-exchanged into 1X PBS using 150 kDa cutoff concentrators (Apollo Biosciences, Bangalore, India). The purity and quantity of the samples were confirmed via sodium dodecyl-sulfate polyacrylamide gel electrophoresis (SDS-PAGE).

### 2.3. Transduction Assay

Pro5 and Lec2 cells were cultured in Minimum Essential Medium Alpha 1 GlutaMax (Gibco, Waltham, MA, USA) containing 10% FBS and 1% ABAM in 15 cm plates and maintained at 37 °C with 5% CO_2_. 24 h before the assay, the cells were passaged and seeded in 24-well plates. The cells were incubated until ~50% confluency was reached. Using a multiplicity of infection (MOI) of 50,000, AAV vectors were added to the wells. In addition to AAVpo.1 and AAVpo.6, AAV9 and AAV5 were used as controls for Lec2 and Pro5 cells, respectively. All AAVs packaged a luciferase reporter gene. For neutralization assays, HEK293 cells were utilized and seeded in 24-well plates in DMEM, similarly to the CHO cells. Prior to addition of the vectors to the cells, they were preincubated 30 min with a dilution series of pooled human serum, obtained from three patients 6 months after administration of an AAV9 vector. The pooled human serum was generated by mixing the three sera 1:1:1. After 48 h of incubation, the media was discarded, 100 µL of lysis buffer was added to each well and incubated at room temperature for 30 min. 40 µL of the lysed cells were transferred to a 96-well plate for each sample and analyzed with a luciferase assay kit (cat#: E1500, Promega, Madison, WI, USA) as described in the manufacturer’s protocol. The luciferase activity was determined with a Synergy HTX BioTek plate reader (Winooski, VT, USA).

### 2.4. Native Dot Immunoblot Analysis

The native dot immunoblot experiments were conducted using a dot blot manifold system (Schleicher and Schuell, Dassel, Germany). Intact AAVpo.1, AAVpo.6, AAV9 (positive control), and AAV5 (negative control) were attached to nitrocellulose membranes at 1 × 10^9^, 5 × 10^8^, and 2.5 × 10^8^ particles/µL via a vacuum-assisted application. For loading controls, the AAVs were heated to 100 °C for 10 min before they were applied to the membrane. All membranes were blocked with 6% milk in 1X PBS, pH 7.4, for 1 h. For the primary antibody, the human monoclonal antibodies (mAbs) [35] or mAb B1 (ARP, Jacksonville, FL, USA, catalog # 690058) were added to the membrane at a concentration of ~1 µg/mL in 6% milk in 1X PBS, 0.1% Tween20, pH 7.4, followed by incubation for 1 h. The membranes were then washed with 1X PBS, 0.1% Tween20, pH 7.4, and the secondary antibody, anti-human HRP (Abcam, Cambridge, UK, catalog # ab6858) for the human mAbs or anti-mouse-HRP (Cytiva catalog # NA931-1ML) for B1, were added at a dilution of 1:50,000 or 1:3000 in 6% milk in 1X PBS, 0.1% Tween20, pH 7.4, respectively. The membranes were incubated for 1 h, then washed with 1X PBS, 0.1% Tween20, pH 7.4. Finally, the Immobilon™ Chemiluminescent Substrate (Millipore, Burlington, MA, USA) was added to the membranes, with the signal detected and recorded on X-ray film (Agfa, Mortsel, Belgium, 8x10 in. 100 NIF) with an average exposure time of 15 s.

### 2.5. Cryo-EM Sample Preparation and Data Collection

Three microliters of AAVpo.1 and AAVpo.6 capsids were applied to glow-discharged C-flat holey carbon-coated grids (Protochips Inc., Cary, NC, USA) and vitrified using a Vitrobot Mark IV (Thermo Fisher) automatic plunge system. The samples were incubated on the grids at 4 °C and 95% humidity for ~3 s, blotted with filter paper, then rapidly plunged into liquid ethane for vitrification. Prior to data collection, all grids were stored at liquid nitrogen temperatures. Grid screening for particle distribution and ice quality was performed in-house (UF ICBR EM Core) using an FEI Tecnai G2 F20-TWIN (FEI) microscope at 200 kV under low-dose conditions (~20 e^−^/Å^2^). High-resolution data was collected at Stanford’s SLAC Cryo-EM Center using a Titan Krios (FEI) electron microscope equipped with a Falcon 4 direct electron detector (Thermo Fisher). The Titan Krios was operated at 300 kV, and a total of 50 movie frames were collected per micrograph with a total electron dose of ~50 e^−^/Å^2^.

### 2.6. Cryo-EM Data Processing and 3D-Image Reconstruction

Three-dimensional maps of AAVpo.1 and AAVpo.6 were reconstructed using the cisTEM software package (version 1.0.0) [36]. Micrographs were imported, followed by a contrast transfer function (CTF) estimation, where poor quality micrographs were identified and eliminated. Particles were automatically picked based on a maximum particle radius of 132 Å and a characteristic radius of 123 Å. The selected particles were classified into 50 2D classes, and only classes that resembled icosahedral capsids were selected. Default settings were used for both the following ab initio 3D reconstruction and automatic refinements steps. Ab initio 3D reconstruction generated a low-resolution map by utilizing 10% of the total boxed particles with imposed icosahedral symmetry. Then, the automatic refinement used the entire data set in addition to the ab initio generated map. For map sharpening, a pre-cutoff B-factor value of −90 Å^2^ was used with variable post-cutoff B-factor values of 0, 20, and 40 Å^2^. All sharpened maps and densities were analyzed in the UCSF-Chimera software (version 1.17.3), and the −90 Å^2^/20 Å^2^ maps were selected for both AAVpo.1 and AAVpo.6 for model building and structure building. The final resolutions of the porcine AAV structures were estimated based on the standard Fourier shell correlation threshold of 0.143 (Table 1).

### 2.7. Model Building and Refinement

In silico model predictions for the AAVpo.1 (NCBI accession number: FJ688147) and AAVpo.6 (NCBI accession number: JX896664) VP3 monomers were generated using the AlphaFold 3 server (https://deepmind.google/science/alphafold/, accessed on 13 November 2024), based on their primary amino acid sequences [37]. 60-mer capsid models (60 copies of VP3) were generated using the online VIPERdb oligomer generator [38]. The 60-mer capsid models were docked into their respective cryo-EM density maps by using the “Fit in Map” tool in UCSF-Chimera [39]. Pixel sizes were optimized to maximize the correlation coefficient, and the maps were resized using the EMAN2 subroutine e2proc3d.py based on the optimized pixel size determined in UCSF-Chimera. The resized maps were converted to the CCP4 format using MAPMAN. Coot was used to manually build and refine the main and side chains of the AlphaFold 3 models using the real-space refinement tool [40]. Additional structural validation in Coot was assessed via the Ramachandran plots, rotamer analysis, and density fitting. PHENIX further refined the models, generating the final refinement parameters and statistics (Table 1) [41].

### 2.8. Comparison of Capsid Structures

For the structural comparison, the monomers of AAVpo.1 and AAVpo.6 to AAV5 (PDB ID: 7KP3) and AAV9 (3UX1) were all superposed in Coot. The monomer structure alignment was visualized using PyMOL (version 3.1, Schrödinger, New York, NY, USA). For the sequence alignments, AlignX in VectorNTI was used to compare the VP1 sequences of the AAVs.

## 3. Results and Discussion

### 3.1. The Porcine AAVs Form Icosahedral Capsids

**Figure 1:**
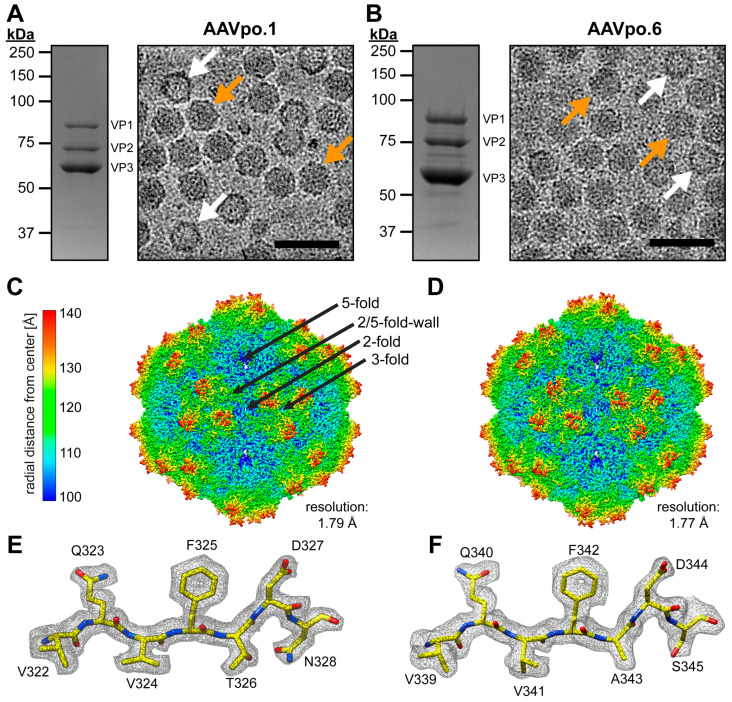
Determination of the AAVpo.1 and AAVpo.6 capsid structure. (**A**,**B**): Left. The SDS-PAGE for purified AAVpo.1 and AAVpo.6 shows the VP1, VP2, and VP3 bands at ~85, 73, and 62 kDa at a ~1:1:10 ratio Right. Cryo-EM micrographs with intact capsids of ~25 nm in diameter are shown. Orange arrows indicate genome-containing (full) capsids, and white arrows point to empty capsids. (**C**,**D**): Capsid electron density maps of AAVpo.1 and AAVpo.6 determined via cryo-EM reconstruction and radially colored (blue to red) based on the distance to the particle center, as shown on the scale bar to the left. The resolution of the structure is calculated based on the standard Fourier shell correlation threshold of 0.143. The map of AAVpo.1 is annotated with the 2-fold, 3-fold, 2/5-fold wall, and 5-fold symmetry axes (black arrows). (**E**,**F**): Amino acid residues 322–328 (339–345) are modeled in the AAVpo.1 (AAVpo.6) density map (gray mesh), contoured at 2σ. The amino acid residues are shown as stick representation and colored according to atom type: C = yellow, O = red, and N = blue. This figure was generated in UCSF-Chimera.

The purified AAVpo.1 and AAVpo.6 samples produced in HEK293 cells exhibited the VP1, VP2, and VP3 capsid protein bands at the expected sizes with their characteristic ~1:1:10 ratio (Figure 1A,B). Cryo-EM micrographs of both samples reveal intact particles of ~25 nm in diameter, comprising a majority of full (DNA containing) and some empty capsids which could be differentiated by their dark (full) and light (empty) appearances. Due to their purity and concentration, the samples were deemed suitable for high-resolution cryo-EM data collection. As previous capsid structures have not shown significant differences between empty and full capsids [28,42], 3D-image reconstructions were conducted on the entirety of the particles regardless of their packaging status. For AAVpo.1 and AAVpo.6 capsid structures were generated from 512,823 and 539,729 particles, resulting in final maps with resolutions of 1.79 and 1.77 Å, respectively (Figure 1C,D). Currently, only two other AAV capsid structures (AAV-DJ, 1.56 Å resolution and AAV9P31 1.76 Å resolution) with a higher resolution than AAVpo.1 and AAVpo.6 have been described [43,44]. It should be noted that the data was possibly resolution-limited by the utilized pixel size of 0.83 Å during data collection, which would theoretically limit the final resolution to 1.66 Å resolution based on the Nyquist limit. The AAVpo.1 and AAVpo.6 structures show the typical capsid morphology of the AAVs with channels at the 5-fold symmetry axes, protrusions surrounding the 3-fold axes, depressions at each 2-fold axis and around the 5-fold pore, only interrupted by the 2/5-fold wall [12]. The amino acid sequence could be readily built to the density maps, starting from amino acid 198 for AAVpo.1 and 217 for AAVpo.6 (Figure 1E,F) which is comparable to all previous AAV capsid structures (Figure S1) [9]. The lower residue number for AAVpo.1 is due to large deletions in the VP1/2 common region. The high resolution enabled the correct placement of all carbonyl groups in the main chain and selections of the right rotamer for the amino acid side chains. Similar to other sub-2Å maps [45], ‘donut holes’ in the aromatic rings were observed for these structures (Figure 1E,F).

### 3.2. AAVpo.1 Is AAV5-like and AAVpo.6 Is Similar to AAV9

**Figure 2:**
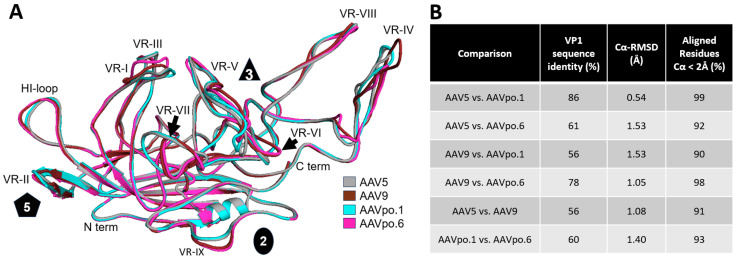
Structural alignment of the VP3 monomers of AAVpo.1, AAVpo. 6, AAV5, and AAV9. (**A**): Superposition of AAV5 (gray) and AAV9 (brown) with AAVpo.1 (cyan) and AAVpo.6 (pink). Variable regions (VR-I to VR-IX), N- and C-termini, and the symmetry axes (2-fold, 3-fold, 5-fold) are annotated. (**B**): Table for the pairwise comparisons of the different AAVs providing the VP1 amino acid sequence identity, the Cα RMSD, and the percentage of aligned residues within a Cα distance of 2 Å.

The determined VP structures for AAVpo.1 and AAVpo.6 conserve the core β-strand A, the eight-stranded antiparallel β-barrel (βB-βI), and α-helix A, similar to all previously determined AAV capsid structures. When structurally aligned with previously determined capsid structures, such as those of AAV5 and AAV9 [46,47], the capsid cores are nearly perfectly superposable (Figure 2A). However, structural differences were observed in the surface loops, the only regions where the Cα positions of the aligned amino acids of the VP structures exceeded a distance of more than 2 Å. These regions were VR-I, -III, -IV, -V, -VI, -VII, the HI-loop, and VR-IX. VR-IV contains the greatest structural differences among the VRs. In contrast, VR-VIII shows a high degree of structural similarity despite low sequence conservation of the loop. Amongst the AAVs compared, AAV5 and AAVpo.1 share the highest percentage of aligned residues (99%) and lowest overall Cα-RMSD value (Figure 2B). Both AAVs share very similar loop conformations in VR-I, -VII, the HI-loop, and VR-IX. On the other hand, AAVpo.6 is more similar to AAV9, with 98% aligned residues; however, significant differences such as in VR-IV, where AAVpo.6 has a 7-amino-acid deletion, resulted in an overall Cα-RMSD of >1 Å. The two porcine AAVs show little similarity in the surface loop structure and are as different to each other as AAV5 is to other AAVs that have been isolated from human tissues [26,48].

### 3.3. AAVpo.1 and AAVpo.6 Are Weak Galactose Binders

**Figure 3:**
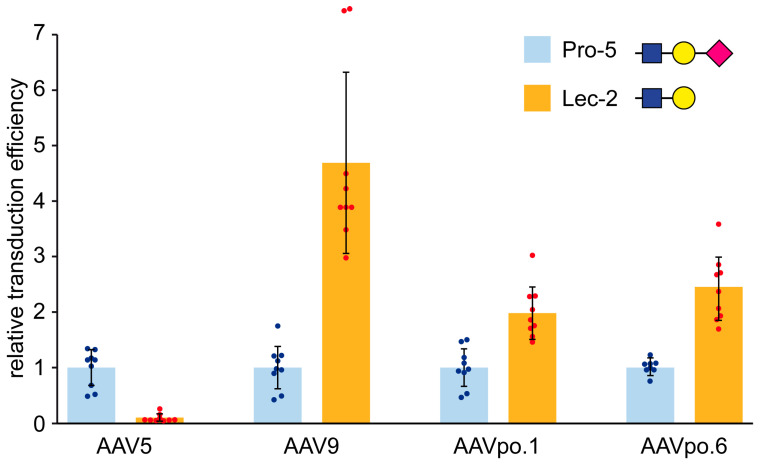
The AAVpo.1 and AAVpo.6 capsids bind galactose. Transduction efficiencies were assessed in Pro-5 (light blue) and Lec-2 (orange) cells, which predominantly present terminal galactose and terminal sialic acid glycans on their cell surfaces, respectively. AAV9 (galactose-binding) and AAV5 (sialic acid-binding) serve as controls. All results are normalized to the transduction efficiency of the Pro-5 cells. Experiments were performed in biological triplicate (with three technical repeats), and error bars represent standard deviations. The individual data points are indicated as blue or red circles, respectively. The glycans (top right) are shown in the symbol representation: pink diamond: N-Acetylneuraminic acid/sialic acid; yellow circle: galactose; and blue square: N-Acetylglucosamine.

Glycans are complex carbohydrates present on cell membranes, involved in cell recognition, signal transduction, and pathogen interactions [49]. Many AAVs, exploit glycan recognition for cellular attachment, with different serotypes exhibiting distinct binding preferences that influence tissue tropism [15]. Sialic acid, a negatively charged glycan molecule, serves as a glycan receptor for AAV1, AAV4, AAV5, and AAV6; heparan sulfate proteoglycan (HSPG) for AAV2, AAV3, AAV6, and AAV13; while neutral galactose is preferred by AAV9 [50,51]. To identify the glycan binding preferences of AAVpo.1 and AAVpo.6, their transduction efficiency was tested in presence of heparin and in Chinese hamster ovary (CHO) Pro-5 and Lec-2 cells which possess alternative cell surface glycan profiles. Heparin did not alter the transduction efficiency of AAVpo.1 and AAVpo.6 indicating that these capsids are not HSPG binders. The Lec-2 cells are a variant of Pro-5 cells with a defect in transport of CMP-sialic acid into the Golgi apparatus, affecting sialylation of glycoproteins [52]. This affects its glycosylation patterns, resulting in galactose being the dominant terminal carbohydrate. AAV9 is a known galactose binder and AAV5 a sialic acid binder, which were used as controls [53]. Thus, as expected, AAV9 showed a ~5-fold increase in transduction efficiency in Lec-2 compared to Pro-5 cells (Figure 3). Vice versa AAV5 performed ~10x better in Pro-5 as compared to Lec-2 cells. AAVpo.1 shows a ~2-fold higher transduction efficiency in Lec-2 cells as compared to Pro-5, indicating a preference for terminal galactose. This behavior is surprising, considering the high sequence identity and structural similarity of AAVpo.1 to AAV5. The sialic acid binding site on the AAV5 capsid has been identified previously and the substitutions of methionine (M) 569 to valine (V) and leucine (L) 587 to threonine (T) on the AAV5 capsid resulted in the loss of sialic acid binding ability [54]. These residues are not conserved in the AAVpo.1 capsid explaining its inability to bind sialic acid compared to AAV5. The AAVpo.6 demonstrated an even larger preference for galactose binding, roughly 3-fold higher in Lec-2 as opposed to Pro-5 cells. Similar—but weaker than AAV9 galactose binding behavior—has previously been observed also for AAVrh.10 and quail adeno-associated virus [25,55]. The suggested amino acids mediating terminal galactose binding in AAV9 are N470, D271, N272, Y446, and W503 [56]. AAVpo.6 shares four of these residues except for N470, which is substituted to serine (S460), whereas only N272 and Y446 are conserved in AAVpo.1. This offers a potential explanation as to why AAVpo.6 exhibits stronger galactose binding characteristics compared to AAVpo.1. However, further research will need to be conducted on AAVpo.1 and AAVpo.6’s glycan binding preferences before final conclusions can be made.

### 3.4. AAVpo.1 and AAVpo.6 Evade Most Anti-AAV9 Antibodies

**Figure 4:**
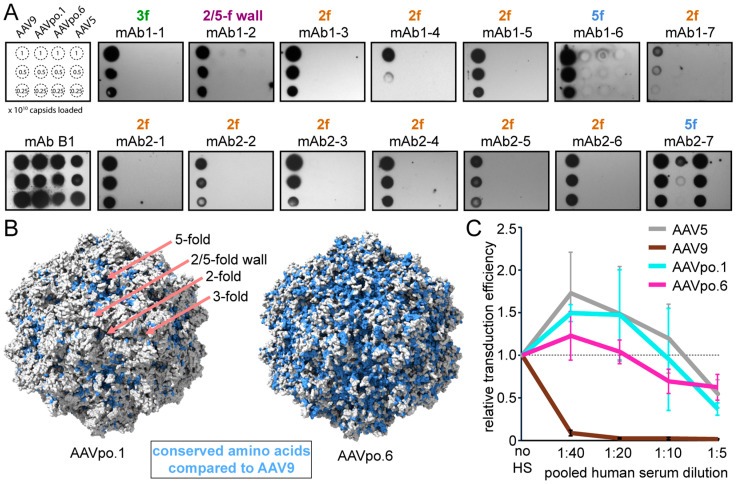
Antigenicity of AAVpo.1 and AAVpo.6. (**A**): Native-immuno dot blots for AAV9, AAVpo.1, AAVpo.6, and AAV5 are shown, screened against mAbs derived from two Zolgensma treated patients. Their binding region to the AAV9 capsid is indicated above. mAb B1 is used as a loading control, and a key for the capsids and quantities loaded shown on the top left. AAVpo.1 escapes 13 out of 14 human antibodies tested, showing partial antibody and capsid binding for mAb2-7. AAVpo.6 escapes 12 out of 14 human antibodies, with strong antibody-capsid binding for mAb2-7 and weak binding to mAb1-6. (**B**): Surface representations of the AAVpo.1 and AAVpo.6 capsids are shown. The surface amino acids that are conserved when compared to AAV9 are colored blue. The positions of the 2-fold (2f), 3-fold (3f), 2/5-fold wall, and 5-fold (5f) symmetry axes (salmon-colored arrows) are shown for the AAVpo.1 capsid. (**C**): Neutralization assay for AAV5, AAV9, AAVpo.1, and AAVpo.6 vectors, carrying luciferase reporter genes, against a pooled human serum obtained from 3 patients 6 months after administration of an AAV9 vector. The determination of the transduction efficiency was conducted in HEK293 cells and is shown relative to the transduction in absence of the human serum. Experiments were performed in biological triplicate (with three technical repeats), and error bars represent standard deviations.

To assess the neutralizing antibody (NAb) escape potential of AAVpo.1 and AAVpo.6, they were tested against a panel of NAbs derived from two post-Zolgensma treated patients [35]. The epitopes of these antibodies were mapped previously, covering key AAV surface symmetry features, namely, the 2-fold depression, 3-fold protrusions, 2/5-fold wall, and 5-fold pore [35,57]. The AAV9 capsid served as positive control since the antibodies were generated in response to exposure to an AAV9 vector, while the AAV5 functioned as a negative control. Serial dilutions of capsids were used, and the B1 antibody, which binds to the denatured C-termini of most AAV capsids [58], served as a sample loading control. Complete escape was observed for all 2-fold, 3-fold, and 2/5-fold wall antibodies amongst the porcine AAVs (Figure 4A).

The VR-IV variations in the porcine capsids relative to AAV9 (Figure 2A) most likely mediate the escape of AAVpo.1 and AAVpo.6 from mAb 1-1, as it binds at the 3-fold symmetry axis. For mAb1-2, VR-I is part of the epitope which also displays structural variability in the porcine capsids compared to AAV9; thus, the antibody is unable to bind the 2/5-fold wall of either capsid. None of the 2-fold binding antibodies bound to AAVpo.1 and AAVpo.6. For the AAVpo.1 capsid, this is not surprising as there is little sequence conservation with the AAV9 residues around the 2-fold region (Figure 4B). In contrast, AAVpo.6 shares many amino acids around the 2-fold depression. For the AAV9 capsid, an escape variant was generated that included the amino acid substitutions: T491R, N562Y, and Y706D [57]. The equivalent residues in the AAVpo.6 capsid are K481, D552, and N696, explaining why the 2-fold antibodies did not recognize the capsid. Lastly, the two 5-fold binding antibodies mAb1-6 and mAb2-7 show some cross-reactivity with the porcine AAV capsids (Figure 4A). Due to the higher similarity of AAVpo.6 in the 5-fold region, both antibodies bound the capsid, but mAb1-6 only with lower affinity. In contrast, AAVpo.1 is not recognized by mAb1-6 and weakly by mAb2-7. As previously shown, AAV5 was not detected by any of the antibodies. Finally, to achieve a broader overview of the capsids’ antigenicity, a neutralization assay was conducted with pooled human sera from patients 6 months after receiving an AAV9 vector (Figure 4C). At all tested dilutions (1:40 to 1:5), AAV9 vectors were completely neutralized, as AAV9 gene therapy recipients have been described to possess very high anti-AAV9 antibody titers [35]. In contrast, the transduction efficiency for AAV5, AAVpo.1, and AAVpo.6 is slightly enhanced at the higher dilutions, possibly due to interactions with serum proteins, as previously shown [59]. At the 1:5 dilution, a transduction efficiency level of ~40–60% compared to the absence of human sera is maintained for these vectors, indicating an overall similar antigenicity for the AAV5, AAVpo.1, and AAVpo.6 capsids.

### 3.5. AAVpo.1 and AAVpo.6 Form Two Distinct Clades

**Figure 5:**
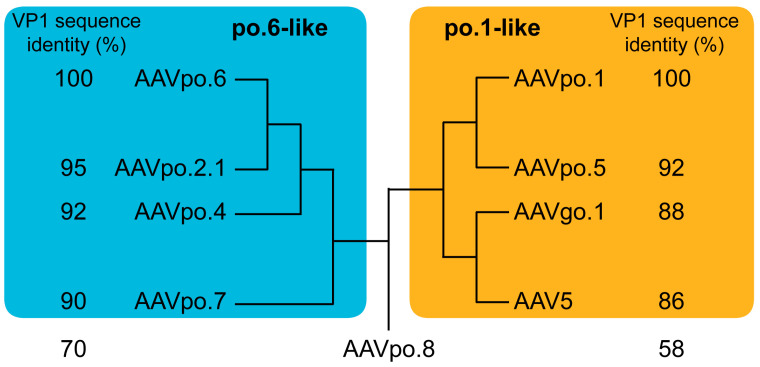
Cladograms of the porcine AAV VP1 sequences. AAVpo.6 and AAVpo.1 form two clusters of AAV sequences. The po.6-like cluster includes AAVpo.6, -po.2.1, -po.4, and -po.7, and the VP1 sequence identity is shown relative to AAVpo.6. The po.1-like AAVs include AAVpo.1, -po.5, -go.1, and AAV5, and their VP1 sequence identities are shown relative to AAVpo.1. The remaining porcine isolate AAVpo.8 is sequence-wise more distant to the two clusters. Its sequence identity is shown to AAVpo.6 (**left**) and AAVpo.1 (**right**).

Multiple AAV isolates from pigs have been described previously [30,32]. Currently, seven complete porcine AAV VP1 protein sequences are deposited in the NCBI databank (AAVpo.1, -po.2.1, -po.4, -po.5, -po.6, -po.7, and -po.8). When the VP1 sequences are compared to each other, AAVpo.6 clusters together with AAVpo.2.1, -po.4, and -po.7 which are within 90% sequence identity (Figure 5). Amino acid sequence differences to AAVpo.6 for these AAVs are found primarily in the VRs. For AAVpo.2.1, three amino acids differ in VR-IV, six in VR-IV, one in VR-VI, seven in VR-VII, ten in VR-VIII, and six in the HI-loop. AAVpo.4 possesses three amino acids differences in VR-I (including one deletion), one in VR-II and -III each, seven in VR-IV and -V each, one in VR-VI, ten in VR-VII, eleven in VR-VIII, ten in the HI-loop, and three in VR-IX. Similarly, AAVpo.7 differs in eleven amino acids differences in VR-I (including one deletion), two in VR-II, one in VR-III, four in VR-IV, six in VR-V each, one in VR-VI, four in VR-VII, ten in VR-VIII, three in the HI-loop, and two in VR-IX. Among the remaining porcine isolates, AAVpo.1 is similar to AAVpo.5 with 92% sequence identity (Figure 5). AAVpo.5 has six amino acid differences in VR-I, two in VR-II, three in VR-III, five in VR-IV, ten in VR-V, two in VR-VI, six in VR-VII, ten in VR-VIII, five in the HI-loop, and one in VR-IX. As described above AAV5 is structurally very similar to AAVpo.1 and thus was included among the po.1-like cluster. Another virus isolate that would belong to this group is AAVgo.1 with 88% sequence identity. Its structure was recently determined [60], and when compared to the AAVpo.1 VP structure, it results in an overall Ca-RMSD of 0.60 Å, comparable to AAV5 (Figure 2B). The remaining porcine AAV, AAVpo.8, is, based on the VP1 sequence identities, 70% to AAVpo.6 and 58% to AAVpo.1, more different than other non-porcine AAVs, including AAV9 (78% to AAVpo.6). Despite this, AAVpo.6 is the closest-related VP1 sequence in the database. Additionally, it displays several insertions or deletions in multiple VRs compared to AAVpo.6 and thus is an interesting candidate for future capsid structure determination. A previous publication suggested the clade identifier G for the AAVpo.1-like members, H for the AAVpo.6-like viruses, and I for the AAVpo.8-like as an extension for the clade classification of the primate AAVs [26,30].

## 4. Conclusions

AAVs have been extensively studied as promising vectors to treat a wide range of monogenic diseases. However, the pre-existing immunity to AAVs in a large portion of the human population remains a challenge. Therefore, AAVs derived from non-human sources are a potential solution to evade the pre-existing neutralizing antibodies. However, an obstacle to using non-human AAVs is that many of the AAVs are unable to transduce mammalian cell lines such as HEK293 cells. Hence, the porcine AAVs hold potential as AAV vectors due to their ability to transduce mammalian cell lines and some of them being able to cross the BBB. The capsid structures determined in this study allow further characterization and annotation of the porcine AAVs. They offer another starting point for future capsid engineering (by rational design, directed evolution, or machine learning approaches), as both AAVpo.1 and AAVpo.6 maintain the general morphologies shared among all AAVs despite differences in the surface loops. In addition to the ability to cross the BBB, the porcine AAVs share another characteristic with AAV by binding terminal galactose glycans but, unlike AAV9, escape most of the human-derived anti-AAV9 mAbs.

## Figures and Tables

**Table 1:** Cryo-EM data collection, image processing, and refinement statistics.

Parameters	AAVpo.1	AAVpo.6
Total number of micrographs	7632	6192
Defocus range (µm)	0.8–2.2	0.8–2.2
Electron dose (e^−^/Å^2^)	50	50
Pixel size (Å/pixel)	0.83	0.83
Number of particles	512,823	539,729
Final map resolution (Å)	1.79	1.77
EMDB accession number	49,042	49,739
**PHENIX model refinement statistics**		
Map correlation coefficient	0.81	0.89
RMSD Bonds (Å)	0.01	0.01
RMSD Angles (°)	0.91	0.95
All-atom clash score	3.47	3.44
**Ramachandran plot (%)**		
Favored	98.3	98.0
Allowed	1.7	2.0
Outliers	0.0	0.0
Rotamer outliers	0.0	0.0
No. of Cβ deviations	0	0
PDB accession number	9N5X	9NRP

## Data Availability

The AAVpo.1 and AAVpo.6 cryo-EM reconstructed density maps and models built for their capsid were deposited in the Electron Microscopy Data Bank (EMDB) and Protein Data Bank (PDB) with the accession numbers/PDB IDs EMD-49042/PDB ID 9N5X (AAVpo.1) and EMD-49739/PDB ID 9NRP (AAVpo.6).

## Supplementary Materials

The following supporting information can be downloaded at: https://www.mdpi.com/article/10.3390/v17091260/s1, Figure S1: VP3 amino acid sequence alignment of AAV5, AAV9, AAVpo.1 and AAVpo.6. The residue numbers indicated above for every 10 amino acids in sequence are based on AAV9 VP1 numbering. The secondary structures are annotated, with β-strands and α-helices indicated (solid black arrows and black rectangles respectively). Dotted lines mark variable regions (VRI-IX) and the HI-loop. Conserved residues for the presented AAVs are predominantly clustered in the β-sheet core (A–I) and shown in yellow. Blue denotes when only one presented AAV is divergent at that position.
