# Supplementary material for: Structural and Functional Characterization of Porcine Adeno-Associated Viruses
Source: Viruses. 2025 Sep 18;17(9):1260. doi: 10.3390/v17091260 (PMC12474171; doi:10.3390/v17091260)
Supplement: Supplementary file 1 [file viruses-17-01260-s001.zip › viruses-3833877-supplementary.pdf]

Supplemental material:

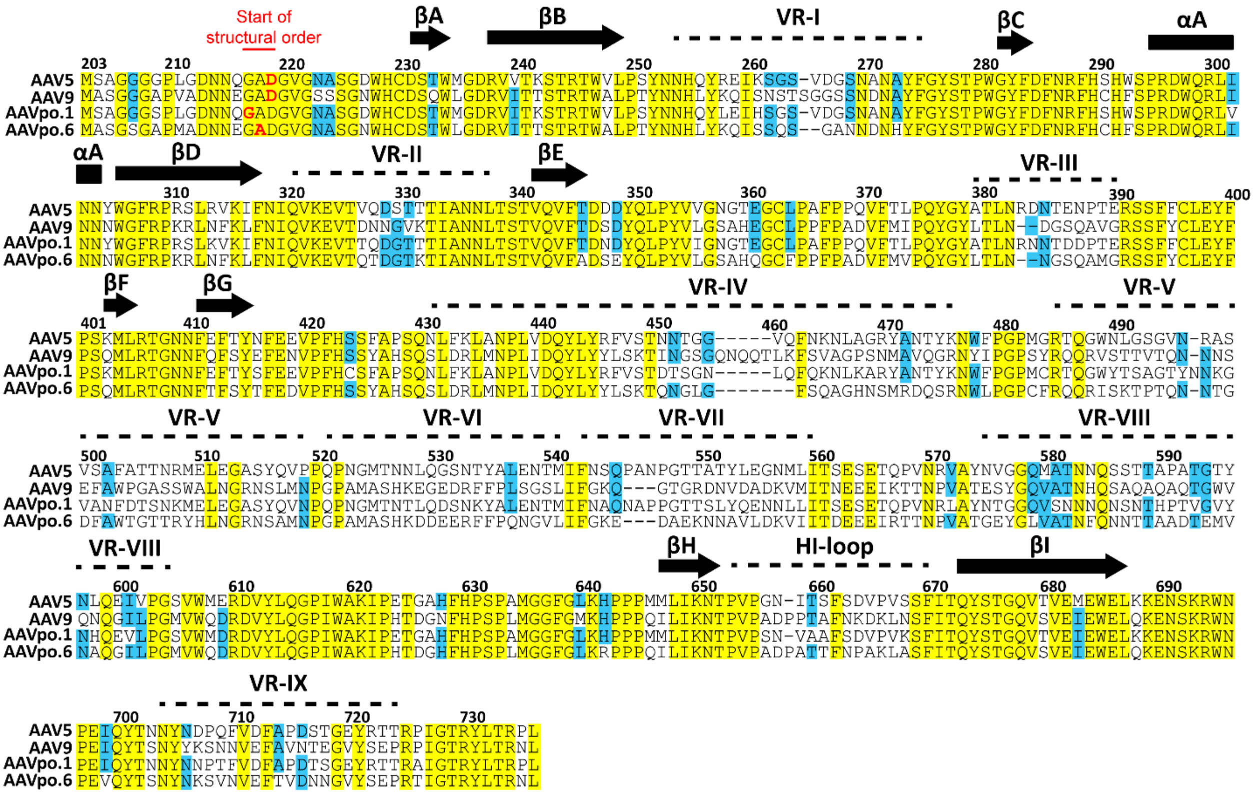

Figure S1: VP3 amino acid sequence alignment of AAV5, AAV9, AAVpo.1 and AAVpo.6. The residue numbers indicated above for every 10 amino acids in sequence are based on AAV9 VP1 numbering. The secondary structures are annotated, with β-strands and α-helices indicated (solid black arrows and black rectangles respectively). Dotted lines mark variable regions (VRI-IX) and the HI-loop. Conserved residues for the presented AAVs are predominantly clustered in the β-sheet core (A–I) and shown in yellow. Blue denotes when only one presented AAV is divergent at that position.
